# Supplementary material for: Quantitative cellular-resolution map of the oxytocin receptor in postnatally developing mouse brains
Source: Nat Commun. 2020 Apr 20;11:1885. doi: 10.1038/s41467-020-15659-1 (PMC7171089; doi:10.1038/s41467-020-15659-1)
Supplement: Supplementary file 1 — Supplementary Information [file 41467_2020_15659_MOESM1_ESM.pdf]

Supplementary Information

Quantitative Cellular-Resolution Map of the Oxytocin Receptor in Postnatally Developing Mouse Brains

Newmaster et al.,

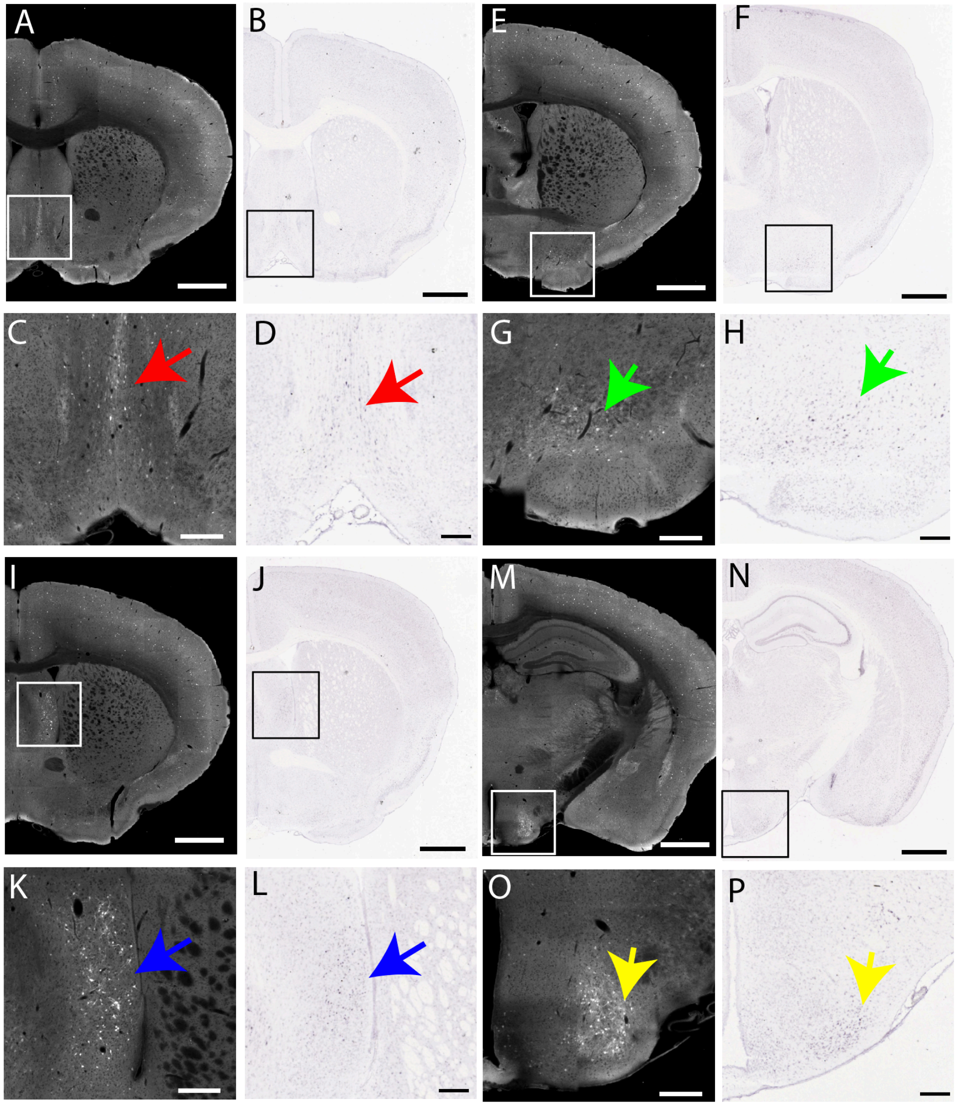

Supplementary Figure 1. Comparison between Venus expression from *Otr-Venus* mice and *Otr* mRNA *in situ* at adult age.

Venus expression from *Otr-Venus* (left column) and *Otr in situ* result (right column) from Allen in situ database (https://mouse.brain-map.org/experiment/show/75081001) in four different example areas: the medial septum (A-D), the nucleus of diagonal band (E-H), the lateral septum (I-L), and the ventral medial hypothalamus ventral lateral (M-P). The second row from each region is a zoomed-in view of the boxed areas in the first row of pictures. Scale bar for the first row = 1mm and the second row = 200µm. Note the matched pattern between *Otr-Venus* mice and endogenous *Otr* expression from the *in situ* data as highlighted with arrows in each region.

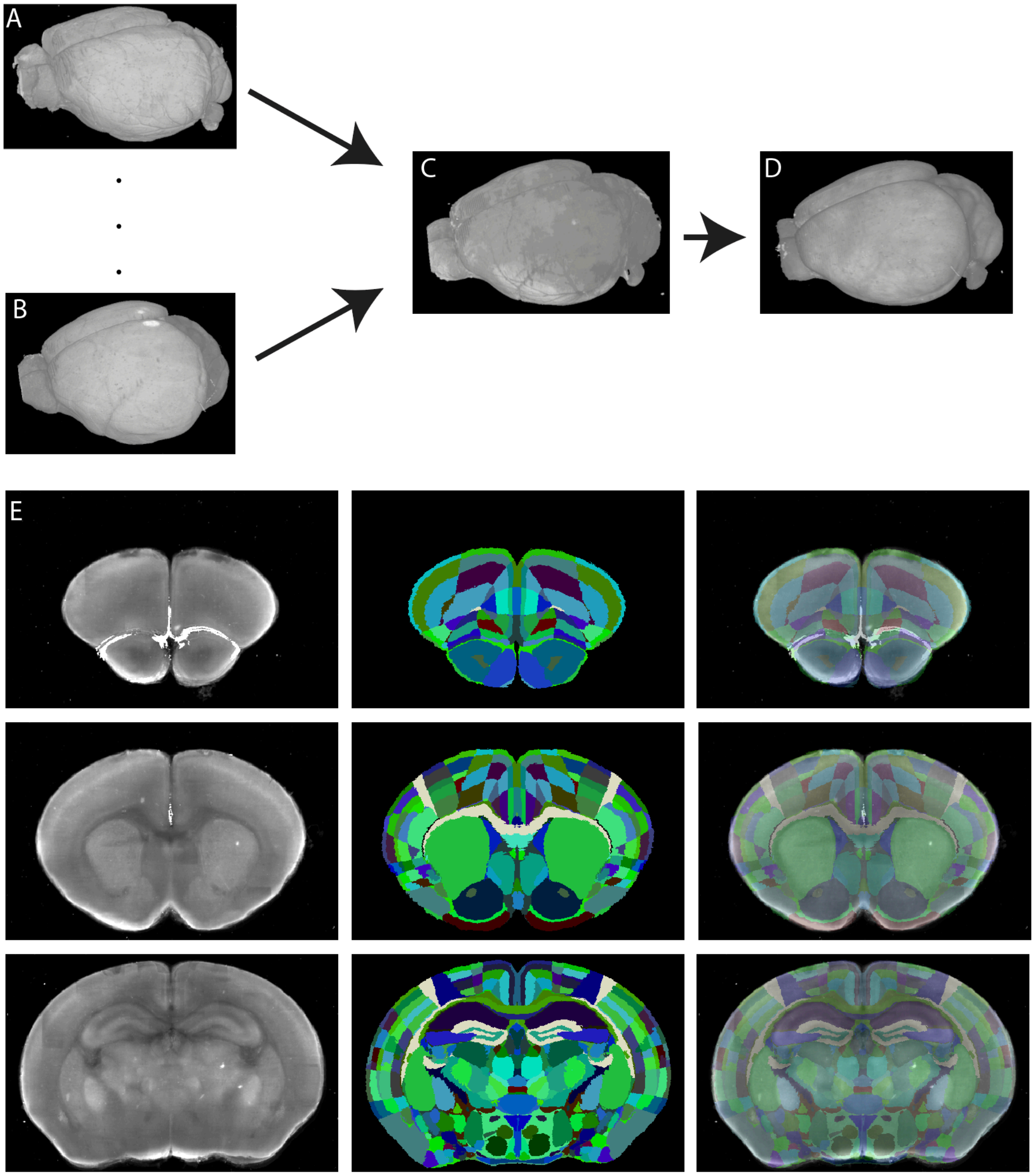

Supplementary Figure 2. Generating age matched template brains and related anatomical labels.

(A-D) Individual 3D brains (A, B) were registered to one best imaged sample (C) from each age group. Registered brains were averaged to generate a template brain (D) at each age. Examples from P21 brains. (E) Examples of anatomical labels from the P7 template brain. Row represents different areas in anterior and posterior axis. The first column is coronal view of the template brain, the second column for registered anatomical labels and the third row for the overlay between the template brain and labels.

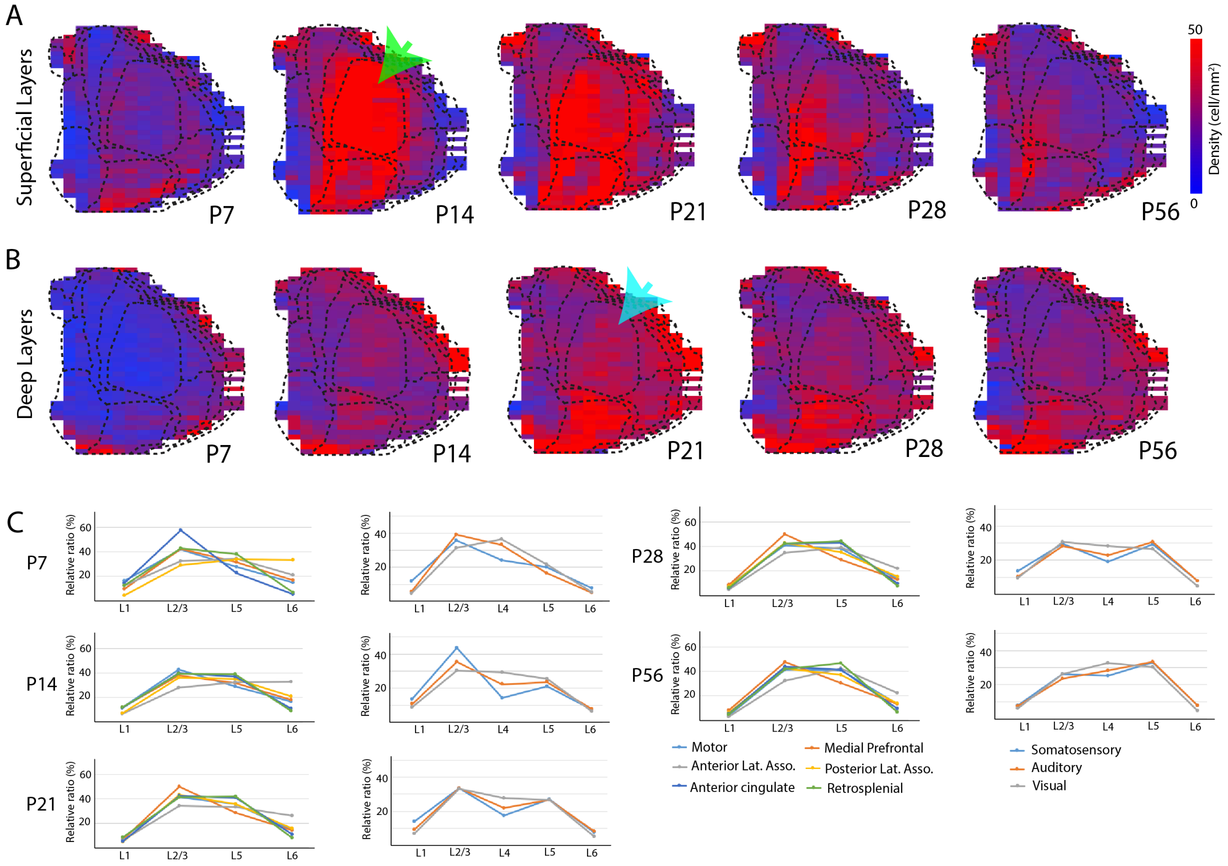

Supplementary Figure 3. OTR expression in the layer specific cortical flatmap.
(A-B) OTR-Venus expression patterns in the 2D cortical flatmap from superficial (A, layer 1-3) and deep (B, layer 5-6) layers at different postnatal ages. The heat map displays the visual representation of density. Note peak OTR density in the somatosensory cortex at P14 in the superficial layer flatmap (green arrow in A) while the peak at P21 in the deep layer flatmap (light blue arrow in B) for temporally heterogeneous OTR expression. (C) Relative densities of OTR-Venus cells across cortical layers in brain regions with motor and associative cortices (left column), and sensory cortices with layer 4 (right column). Density in each layer is normalized by total density of the whole layer in each brain region.

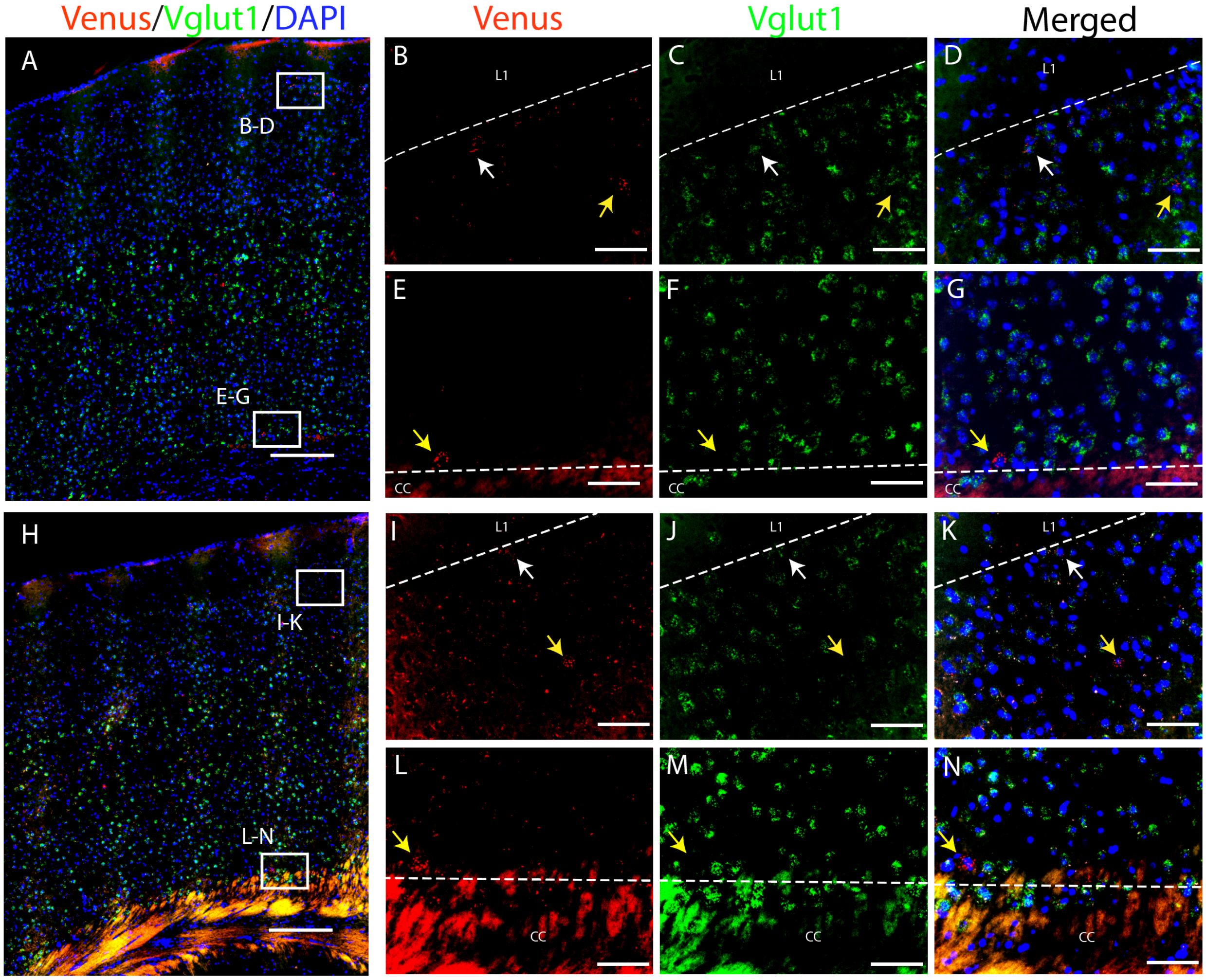

Supplementary Figure 4. *Vglut1* colocalization in the OTR-Venus cortical neurons. (A-N) fluorescent in situ hybridization of *Vglut1* and *Venus* from somatosensory cortical area from P21 (A-G) and P56 (H-N) in $Otr^{Venus/+}$ mice. Scale bar in (A, H) = 200 µm. Examples of high magnification images in the upper layer (B-D, I-K) and the layer 6b (E-G, L-N) from boxed areas in (A, H). Scale bar in (B-G, I-N) = 50 µm. White arrows for *Venus* (+) cells co-expressing *Vglut1*, and yellow arrows for *Venus* (+) cells without *Vglut1* colocalization. Abbreviations: L1 in (B-D, I-K) for the layer 1, cc in (E-G, L-N) for the corpus callosum.

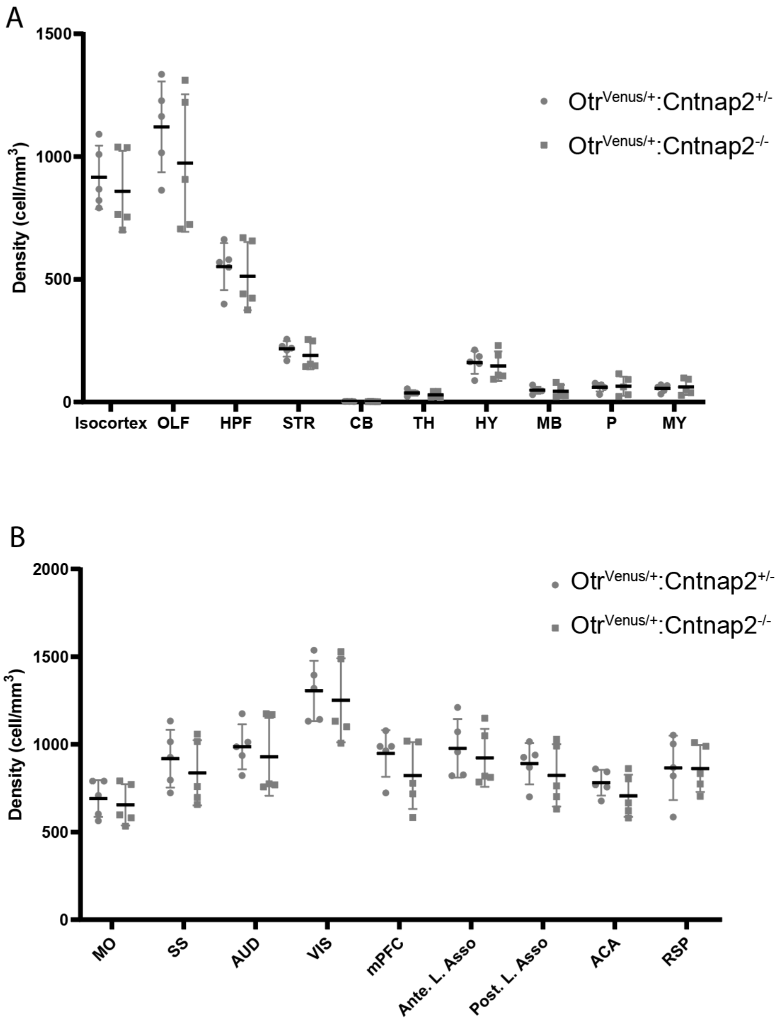

Supplementary Figure 5. OTR expression in *Cntnap2* KO mice.
(A-B) Densities of OTR expressing cells in the major brain regions (A) and isocortical areas (B) between the control (*Otr*$^{Venus/+}$:*Cntnap2*$^{+/-}$) and *Cntnap2* KO (*Otr*$^{Venus/+}$: *Cntnap2*$^{-/-}$) mice. None of areas is significantly different between groups. Abbreviations in (A): OLF (olfactory area), HPF (Hippocampal formation), STR (Striatum), CB (Cerebellum), TH (Thalamus), HY (Hypothalamus), MB (Midbrain), P (Pons), MY (Medulla), Abbreviations in (B): MO (motor), SS (somatosensory), AUD (auditory), VIS (Visual), mPFC (medial prefrontal), Ante. L. Asso. (Anterior lateral association), Post. L. Asso (Posterior lateral association), ACA (Acingulate), RSP (Retrosplenial). Error bars = Mean ± Standard Deviation

	P21			P56		
**Brain area**	**Upper layer**	**Deeper layer**	**Layer 6b**	**Upper layer**	**Deeper layer**	**Layer 6b**
**Medial prefrontal cortex**	61% (593/980)	59% (290/492)	21% (9/43)	68% (345/507)	64% (156/242)	56% (19/34)
**Somatosensory cortex**	71% (362/507)	49% (136/280)	57% (13/23)	71% (494/697)	74% (58/78)	59% (17/29)
**Visual Cortex**	67% (481/715)	47% (310/655)	19% (7/36)	78% (269/209)	77% (245/317)	55% (47/83)

Supplementary Table 1. ***Vglut1*** **colocalization with cortical OTR-Venus neurons.**
Data from the medial prefrontal cortex, the somatosensory cortex, and the visual cortex. Data presented as percentage of colocalized cells (*Otr* and *Vglut1* positive cells/total *Otr* positive cells) in each brain region.
