# Supplementary material for: Quantitative cellular-resolution map of the oxytocin receptor in postnatally developing mouse brains
Source: Nat Commun. 2020 Apr 20;11:1885. doi: 10.1038/s41467-020-15659-1 (PMC7171089; doi:10.1038/s41467-020-15659-1)
Supplement: Supplementary file 3 — Reporting Summary [file 41467_2020_15659_MOESM3_ESM.pdf]

nature research

Corresponding author(s): *Double-blind peer review submissions: write DBPR and your manuscript number here instead of author names.*

Last updated by author(s): *YYYY-MM-DD*

Reporting Summary

Nature Research wishes to improve the reproducibility of the work that we publish. This form provides structure for consistency and transparency in reporting. For further information on Nature Research policies, seeAuthors & Referees and theEditorial Policy Checklist .

Statistics

For all statistical analyses, confirm that the following items are present in the figure legend, table legend, main text, or Methods section.

n/a	Confirmed	
☐	☒	The exact sample size (*n*) for each experimental group/condition, given as a discrete number and unit of measurement
☐	☒	A statement on whether measurements were taken from distinct samples or whether the same sample was measured repeatedly
☐	☒	The statistical test(s) used AND whether they are one- or two-sided<br>*Only common tests should be described solely by name; describe more complex techniques in the Methods section.*
☐	☒	A description of all covariates tested
☐	☒	A description of any assumptions or corrections, such as tests of normality and adjustment for multiple comparisons
☐	☒	A full description of the statistical parameters including central tendency (e.g. means) or other basic estimates (e.g. regression coefficient) AND variation (e.g. standard deviation) or associated estimates of uncertainty (e.g. confidence intervals)
☐	☒	For null hypothesis testing, the test statistic (e.g. *F*, *t*, *r*) with confidence intervals, effect sizes, degrees of freedom and *P* value noted<br>*Give P values as exact values whenever suitable.*
☒	☐	For Bayesian analysis, information on the choice of priors and Markov chain Monte Carlo settings
☒	☐	For hierarchical and complex designs, identification of the appropriate level for tests and full reporting of outcomes
☐	☒	Estimates of effect sizes (e.g. Cohen's *d*, Pearson's *r*), indicating how they were calculated

Our web collection on statistics for biologists contains articles on many of the points above.

Software and code

Policy information about availability of computer code

Data collection	Custom built stitching algorithm to reconstruct images from serial two-photon tomography and Elastix for image registration were publicly distributed in the Kim et al., 2017 Cell, DOI: 10.1016/j.cell.2017.09.020). Elastix registration parameter files can be found in Supplementary Data 2.
Data analysis	1. Code to perform following analyses is publicly distributed in the Kim et al., 2017 Cell, DOI: 10.1016/j.cell.2017.09.020).<br>- Open source statistical package R to calculate statistical significance including multiple comparison correction.<br>- Plotting cell distribution in cortical flatmaps including layer specific ones, New layer specific cortical flatmaps are included in the current manuscript.<br>2. Prism 8 (GraphPad) is used to plot individual data distribution

For manuscripts utilizing custom algorithms or software that are central to the research but not yet described in published literature, software must be made available to editors/reviewers. We strongly encourage code deposition in a community repository (e.g. GitHub). See the Nature Research guidelines for submitting code & software for further information.

Data

Policy information about availability of data

All manuscripts must include a data availability statement. This statement should provide the following information, where applicable:

- Accession codes, unique identifiers, or web links for publicly available datasets
- A list of figures that have associated raw data
- A description of any restrictions on data availability

Template brains and associated anatomical labels at different postnatal development is available as Supplementary Data 3.
Layer specific cortical flatmap label files are available as Supplementary Data 4.
Representative high-resolution images of both OTR-Venus and OTR-eGFP can be found in http://kimlab.io/brain-map/OTR/
All full resolution images of Otr-Venus, Otr-eGFP, Otr-Cre:Ai14 mice are freely available to download in the Brain Image Library at ftp://

download.brainimagelibrary.org:8811/56/fb/56fb1b25ca6b5fae/

Field-specific reporting

Please select the one below that is the best fit for your research. If you are not sure, read the appropriate sections before making your selection.

- [x] Life sciences
- [ ] Behavioural & social sciences
- [ ] Ecological, evolutionary & environmental sciences

For a reference copy of the document with all sections, see nature.com/documents/nr-reporting-summary-flat.pdf

Life sciences study design

All studies must disclose on these points even when the disclosure is negative.

Sample size

To identify sexual dimorphism, we performed statistical comparisons between males and females in OTR-Venus cell counts across different anatomical regions using open source statistical package R. We estimated our sample size using the power analysis as performed in our previous publication23. When significance level ($\alpha < 0.05$) and assumed effect size (0.85), we expected that over 80% of anatomical regions reach sufficient power with N = 5 samples per group. For statistical analysis between groups, we assumed the cell counts at a given anatomical area follow a negative binomial distribution and performed statistical analysis as described before 23,26.

To compare OTR cell density of Cntnap2 KO mice, we use N = 5 brains for each genotype.

For dendritic spine density measurement, we use N = 3 brains for each genotype.

Data exclusions

Samples with poor perfusion, poor brain dissection, and/or poor imaging quality were excluded from further analysis

Replication

Our analysis relies on good representation of endogenous oxytocin receptor (OTR) from OTR reporter mice. We examined two existing reporter line (OTR-eGFP and OTR-Venus) and compared their reporter expression to endogenous OTR expression using RNA in situ hybridization as illustrated in Figure 1. Using this validation method, we confirmed that OTR-Venus mice serve as a good reporter line while OTR-eGFP mice contain both false positive and false negative.

Furthermore, we used publicly available in situ gene expression databased from Allen Institute of Brain Sciences. This additional comparison also confirmed that OTR-Venus represents endogenous OTR expression well.

Randomization

Our work does not include data with experimental manipulation requiring randomization. Thus, no randomization was used.

Blinding

When we assess colocalization of fluorescent signals from different target probes, two expert biologists quantified signals independently without knowing other's results. For spine density comparison using Golgi staining, one expert quantify spine density without knowing the genotypes of samples.

Reporting for specific materials, systems and methods

We require information from authors about some types of materials, experimental systems and methods used in many studies. Here, indicate whether each material, system or method listed is relevant to your study. If you are not sure if a list item applies to your research, read the appropriate section before selecting a response.

Materials & experimental systems

n/a	Involved in the study	
☐	☒	Antibodies
☒	☐	Eukaryotic cell lines
☒	☐	Palaeontology
☐	☒	Animals and other organisms
☒	☐	Human research participants
☒	☐	Clinical data

Methods

n/a	Involved in the study	
☒	☐	ChIP-seq
☒	☐	Flow cytometry
☒	☐	MRI-based neuroimaging

Antibodies

Antibodies used

anti-GAD67 monoclonal antibody: Milipore, Cat# MAB5406, Clone 1G10.2, Lot. no. 3216488

Validation

anti-GAD67 monoclonal antibody: Reacts with the 67kDa isoform of Glutamate Decarboxylase (GAD67) of rat, mouse and human origins, other species not yet tested. More information on validation can be found in http://www.emdmillipore.com/US/en/product/Anti-GAD67-Antibody-clone-1G10.2,MM_NF-MAB5406

Animals and other organisms

Policy information about studies involving animals; ARRIVE guidelines recommended for reporting animal research

Laboratory animals	OTR-Venus mice: 129 x C57BL/6J mixed genetic background, both males and females, collected at P7, P14, P21, P28, P56<br>OTR-Cre mice: 129 x C57BL/6J mixed genetic background, used to generate OTR-Cre:Ai14 mice<br>Ai14 mice: C57 background, Jax: 007914, used to generate OTR-Cre:Ai14 mice<br>OTR-Cre:Ai14 mice: 129 x C57BL/6J mixed genetic background, both males and females, collected at P7, P14, P21, P28, P56<br>OTR-eGFP mice: mixed FVB/N x Swiss-Webster background strain, RRID:MMRRC_012844-UCD, both males and females, collected at P7, P14, P21, P28, P56<br>Cntnatp2 KO mice: C57 background, Jax: 017482, used to generate OTR-Venus:Cntnap2 mice
Wild animals	N/A
Field-collected samples	N/A
Ethics oversight	Animal procedures are approved by Florida State University, Tohoku University, and the Penn State University Institutional Animal Care and Use Committee (IACUC).

Note that full information on the approval of the study protocol must also be provided in the manuscript.
