# Supplementary material for: Quantitative cellular-resolution map of the oxytocin receptor in postnatally developing mouse brains
Source: Nat Commun. 2020 Apr 20;11:1885. doi: 10.1038/s41467-020-15659-1 (PMC7171089; doi:10.1038/s41467-020-15659-1)
Supplement: Supplementary file 4 — Description of Additional Supplementary Files [file 41467_2020_15659_MOESM4_ESM.pdf]

Description of Additional Supplementary Files

File Name: Supplementary Data 1
Description: **A list of OTR Densities across different brain regions at different postnatal ages.**
Column A: Abbreviations of brain regions, Column B: Full names, Column C-G: Mean density (cell/mm3) at different postnatal ages. These columns are conditionally formatted with red color to highlight areas with high density. The heatmap color range between 0 (transparent) and 5000 (red). Column H-L: standard deviation at different postnatal ages.

File Name: Supplementary Data 2
Description: **Image registration parameter files for Elastix.**
Par_affine.txt and Par_bspline.txt are parameter files for our linear and non-linear image registration using Elastix, respectively.

File Name: Supplementary Data 3
Description: **Postnatal brain atlases**

File Name: Supplementary Data 4
Description: **Layer specific cortical flatmaps**

File Name: Supplementary Movie 1
Description: Averaged OTR density at P56 (Green) overlaid in an age matched reference brain (Left) and anatomical segmentations (Right)

File Name: Supplementary Movie 2
Description: Averaged OTR density at P28 (Green) overlaid in an age matched reference brain (Left) and anatomical segmentations (Right)

File Name: Supplementary Movie 3
Description: Averaged OTR density at P21 (Green) overlaid in an age matched reference brain (Left) and anatomical segmentations (Right)

File Name: Supplementary Movie 4
Description: Averaged OTR density at P14 (Green) overlaid in an age matched reference brain (Left) and anatomical segmentations (Right)

File Name: Supplementary Movie 5
Description: Averaged OTR density at P7 (Green) overlaid in an age matched reference brain (Left) and anatomical segmentations (Right)
