# Supplementary material for: Quantitative cellular-resolution map of the oxytocin receptor in postnatally developing mouse brains
Source: Nat Commun. 2020 Apr 20;11:1885. doi: 10.1038/s41467-020-15659-1 (PMC7171089; doi:10.1038/s41467-020-15659-1)
Supplement: Supplementary file 7 — Supplementary Data 3 [file 41467_2020_15659_MOESM7_ESM.zip › Postnatal_Atlases/How to use Postnatal atlases.docx]

**How to use Postnatal atlases**

General information

Reference brains at different ages have corresponding anatomical label files. For example, “P7_label.tif” for “P7_brain.tif”.

In the label file, each anatomical region has unique 16bit identifier number. The full anatomical information of each ID can be found in “Label-info.txt”.

All reference brains use the same ID structure based on “Kim_ref_Label-info.txt”.

“Label-info.txt” has information on hierarchical organization in each brain region based on Allen ontology^1^.

Structure order in the “Label-info.txt” helps to see the hierarchical organization.

How to use atlases

Detailed instruction to perform quantitative brain (qBrain) mapping method has been described in our previous publication^2^. Many analysis codes were released together with the previous publication. Readers are strongly encouraged to read the method section of the previous publication.

These atlases have 20 x 20 x 50 µm (*x,y,z*) voxel resolution.

If you have high resolution 3D images, you can down-sample your size first to match the voxel resolution.

Then, you can use Elastix^3^ to perform image registration to fit your brain samples and to map your detected signals to the age matched reference brain.

Reference

1. Sunkin, S. M. *et al.* Allen Brain Atlas: an integrated spatio-temporal portal for exploring the central nervous system. *Nucleic Acids Res.* **41,** D996–D1008 (2013).

2. Kim, Y. *et al.* Brain-wide Maps Reveal Stereotyped Cell-Type-Based Cortical Architecture and Subcortical Sexual Dimorphism. *Cell* **171,** 456–469.e22 (2017).

3. Klein, S., Staring, M., Murphy, K., Viergever, M. A. & Pluim, J. P. W. elastix: a toolbox for intensity-based medical image registration. *IEEE Trans Med Imaging* **29,** 196–205 (2010).
